# Raccoon rabies control and elimination in the northeastern USA and southern Québec, Canada

**DOI:** 10.1017/S095026882300047X

**Published:** 2023-03-22

**Authors:** Amy J. Davis, Marianne Gagnier, Ariane Massé, Kathleen M. Nelson, Jordona D. Kirby, Ryan Wallace, Xiaoyue Ma, Christine Fehlner-Gardiner, Richard B. Chipman, Amy T. Gilbert

**Affiliations:** 1 United States Department of Agriculture, Animal and Plant Health Inspection Service, Wildlife Services, National Wildlife Research Center, Fort Collins, CO, USA; 2 Ministère de l'Environnement, de la Lutte aux changements climatiques, de la Faune et des Parcs, Québec City, QC, Canada; 3 United States Department of Agriculture, Animal and Plant Health Inspection Service, Wildlife Services, National Rabies Management Program, Concord, NH, USA; 4 Centers for Disease Control and Prevention, Atlanta, GA, USA; 5 Centre of Expertise for Rabies, Ottawa Laboratory Fallowfield, Canadian Food Inspection Agency, Ottawa, ON, Canada

**Keywords:** Disease elimination, dynamic occupancy, multi-method occupancy, ORV, rabies virus, raccoon, surveillance, wildlife disease

## Abstract

Rabies virus (RABV) is a deadly zoonosis that circulates in wild carnivore populations in North America. Intensive management within the USA and Canada has been conducted to control the spread of the raccoon (*Procyon lotor*) variant of RABV and work towards elimination. We examined RABV occurrence across the northeastern USA and southeastern Québec, Canada during 2008–2018 using a multi-method, dynamic occupancy model. Using a 10 km × 10 km grid overlaid on the landscape, we examined the probability that a grid cell was occupied with RABV and relationships with management activities (oral rabies vaccination (ORV) and trap-vaccinate-release efforts), habitat, neighbour effects and temporal trends. We compared raccoon RABV detection probabilities between different surveillance samples (e.g. animals that are strange acting, road-kill, public health samples). The management of RABV through ORV was found to be the greatest driver in reducing the occurrence of rabies on the landscape. Additionally, RABV occupancy declined further with increasing duration of ORV baiting programmes. Grid cells north of ORV management were at or near elimination (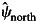 = 0.00, s.e. = 0.15), managed areas had low RABV occupancy (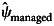 = 0.20, s.e. = 0.29) and enzootic areas had the highest level of RABV occupancy (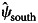 = 0.83, s.e. = 0.06). These results provide evidence that past management actions have been being successful at the goals of reducing and controlling the raccoon variant of RABV. At a finer scale we also found that vaccine bait type and bait density impacted RABV occupancy. Detection probabilities varied; samples from strange acting animals and public health had the highest detection rates. Our results support the movement of the ORV zone south within the USA due to high elimination probabilities along the US border with Québec. Additional enhanced rabies surveillance is still needed to ensure elimination is maintained.

## Introduction

Rabies virus (RABV) is a deadly zoonosis that circulates in wild carnivore populations in North America [1]. After epizootic expansion in the latter end of the twentieth century, the raccoon (*Procyon lotor*) variant of RABV became enzootic in the mid-Atlantic and northeastern USA [2]. The northward spread also resulted in incursions of raccoon RABV across the border into the provinces of Ontario, Québec and New Brunswick, Canada [3]. Two incursions have occurred in Québec, one in 2006 and another in 2015. Intensive management with oral rabies vaccination (ORV) in both instances, along with trap-vaccinate-release (TVR) programmes during the 2006 occurrence, controlled the incursions [3, 4]. Enhanced surveillance (i.e. active surveillance to support raccoon rabies management) along the Québec border with the USA is a crucial part of ensuring the new incursions are detected swiftly for targeted management [4].

Management of the raccoon variant of RABV in the USA is aimed at preventing the spread westwards in the USA and northwards into Canada, and to work towards the elimination along the east coast of the USA [5, 6]. In USA where the raccoon variant of RABV circulates, the overall burden of animal cases is greatest and post-exposure prophylaxis is administered at higher rates compared to states with other RABV variants [7] and spill over of the raccoon variant into other species is elevated compared to skunk RABV variants [8]. These factors coupled with the demonstrated potential for rapid expansion of epizootic regions of the raccoon variant of RABV highlight the need for effective management of this variant. ORV campaigns began in the northeastern USA in the early 1990s using RABORAL V-RG® (V-RG; a registered trademark in the USA and elsewhere of Merial, Inc., now Boehringer Ingelheim, Athens, Georgia). Within Québec, ORV baiting with V-RG was used from 2006 to 2008 [4], but in 2009, a second vaccine bait type began being used called the Ontario Rabies Vaccine Bait (hereafter, ONRAB; Artemis Technologies, Inc., an indirect, wholly owned subsidiary of Ceva Sante Animale, S.A.) [4]. ONRAB was experimentally deployed in the northeastern USA starting in 2012 [9]. Both vaccine baits were typically deployed at 75 baits/km^2^ [4, 10 and 11]. However, bait densities were increased to 150/km^2^ to either coincide with higher raccoon densities in an area or in attempts to achieve seroprevalence levels suggested to suppress transmission (60–80% [12–14]) for control and elimination.

Management of RABV in the northeastern USA is conducted in collaboration with Québec as both the US federal and Canadian provincial governments seek to limit the threat of raccoon rabies in their respective countries and recognise that raccoon rabies has crossed international borders in the past [15]. Previous work has examined raccoon RABV elimination status and potential risk corridors using solely data from the USA [16], which suggested areas along the New York–Vermont border (Lake Champlain valley) and in the western part of Franklin county, New York (St. Lawrence River valley) may pose a greater risk for northward incursions. By including and leveraging data from Québec, we can examine factors related to previous incursions to better help understand risk factors (e.g. distance to recent rabies cases, time since last ORV baiting) and conversely, examine what factors relate to better management success at reducing rabies occurrence (e.g. vaccine bait type, bait density, duration of baiting).

Effective wildlife rabies management requires continuous enhanced surveillance for determination of where rabies is enzootic and where it is absent. Using analytical approaches such as occupancy analysis [17], we can evaluate both the probability of raccoon RABV occurrence in an area and the probability of detecting raccoon RABV when it is present based on the surveillance sampling in the area. We retrospectively evaluate these cross-border surveillance efforts to inform and refine future surveillance efforts in the context of dynamic landscape epidemiology of raccoon RABV. Our objectives for this study are to (1) understand patterns that impact raccoon RABV occurrence in the cross-border region in the northeastern USA and Québec, (2) evaluate the probability of elimination of raccoon RABV across space and time, (3) determine risk corridors, (4) compare surveillance categories and (5) inform future surveillance needs to ensure long-term regional elimination of raccoon RABV along the border of the USA and Québec.

## Methods

### Study area

**Fig. 1.**
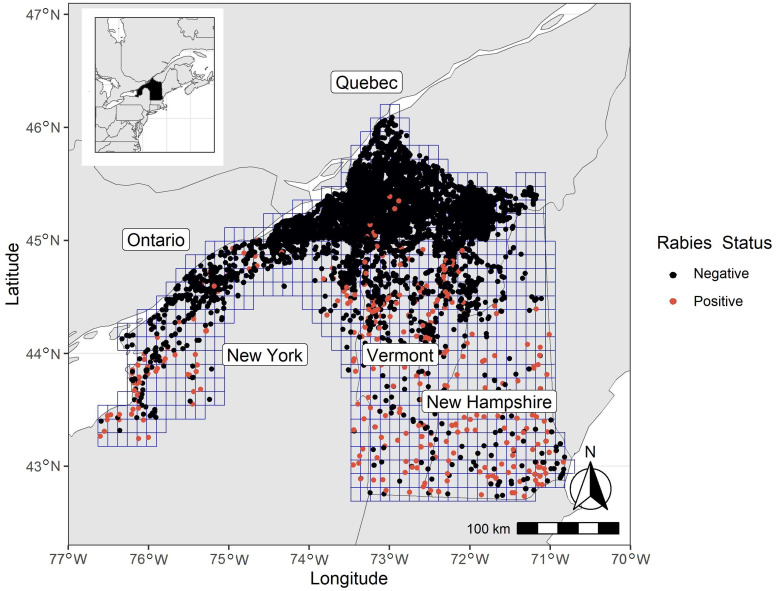
Map of study area for raccoon RABV occupancy analysis in the northeastern USA and in southern Québec, Canada. The 10 km × 10 km grid cells of the study area are shown in blue. The red dots are locations of RABV-positive samples and black dots are locations of RABV-negative samples. These samples are from 2008 to 2018 by all surveillance types.

Our study area encompasses southern regions in Québec, Canada along the US border that have been actively surveilled and managed for raccoon RABV and extends into the northeastern USA to include all of New Hampshire, all of Vermont and northern New York (Fig. 1). The habitat over the entire study area was predominantly deciduous and mixed forest (~49%), with the remainder representing evergreen forests (12%), cultivated crops (18%), pasturelands (1%) and developed areas (6%). The study area in Québec consisted of more cultivated cropland (45%) than the study area in the USA (12%), and less evergreen forest cover (1% in Québec compared to 15% in the USA). The study area included important landscape features such as Lake Champlain, the Adirondack Mountains and the St. Lawrence River. The elevation in the study area ranged from sea level to 1917 m.

### Data

Three RABV surveillance data sources were used for this analysis: enhanced rabies surveillance (ERS) data collected by the US Department of Agriculture, Animal and Plant Health Inspection Service, Wildlife Services (WS), National Rabies Management Program (hereafter, NRMP) from New York, Vermont and New Hampshire; public health surveillance data from New York, Vermont and New Hampshire that are collected from potential cases of human or pet exposures (both ERS and public health data are reported annually to the Centers for Disease Control and Prevention (CDC)) and data collected by the Ministère de l'Environnement, de la Lutte aux changements climatiques, de la Faune et des Parcs from Québec as part of enhanced and public health rabies surveillance. Data used in these analyses spanned 2008–2018 across all sources.

The data collected for each sampled animal include the species, location where the animal was collected, date of collection, agency that collected the sample, how the animal was encountered (e.g. surveillance trapped, road-kill sample) and field comments. Brain tissue from each animal was tested for RABV using either the direct rapid immunohistochemical test [18] or direct fluorescent antibody assay (DFA [19–20]). When possible, DFA-positive samples were typed using discriminatory monoclonal antibody panels [21–22]. Otherwise, cases in raccoons from the study area were assumed to be caused by the raccoon variant of RABV.

The ERS data are categorised based on a combination of the animal's behaviour and the method of sample collection. The categories are: (1) animals observed as sick or strange acting, (2) animals that were found dead (in a yard, woods, etc., but not road-kill), (3) road-kill animals, (4) animals that were trapped and euthanised (by WS) specifically for RABV surveillance, (5) animals sampled that were not for intentional rabies surveillance (e.g. nuisance animals reported by the public that were otherwise healthy, animals caught by the public during furbearer trapping) termed ‘other-known’ and (6) sampling from any unknown method of collection where behaviour was not observed [23]. These classifications were formalised in the USA during 2016, whereas prior to 2016 samples were categorised post-collection based on information in the sample record on the animal's fate, the agency collecting the sample and comments taken during sample collection [24]. A similar approach was used to categorise the ERS samples from Québec. Public health samples were designated as a separate (seventh) surveillance category.

### Analytical methods

To conduct the RABV occupancy analysis, we overlaid a 10 km × 10 km grid across the study area [24, 25]. Within our dynamic occupancy approach, we used seasons as primary sampling periods to accommodate both the incubation and infectious periods of RABV, and any sampling events within a season are considered secondary sampling periods in an occupancy framework. Individual raccoons that were sampled were laboratory-confirmed as RABV positive or negative. When a raccoon was RABV positive, the grid cell in which the raccoon was sampled (and the season it was found) was coded as occupied with raccoon RABV. Grid cells where only negative raccoons were sampled may be occupied with raccoon RABV and surveillance failed to detect RABV, or the grid cell may be absent of raccoon RABV. The raccoon variant of RABV commonly spills over into other species [8]. We used cases of the raccoon variant in other terrestrial mammals to inform the occupancy analysis; however we did not estimate the detection probability of surveillance including the negative case data from species other than raccoons, as they are not the primary reservoir for the raccoon variant of RABV.

We used a dynamic occupancy model to estimate grid and season occupancy across the study area during 2008–2018. We used a multi-method occupancy approach that allows for the separate detection estimation by surveillance sample type [25, 26]. Using a multi-method approach allows for the integration of information learned across different sample types to help inform both the overall occupancy status and the individual detection probability estimates. In a dynamic occupancy model, the transition parameters of local extinction (an area that was occupied becoming unoccupied) and local colonisation (an area that was unoccupied becoming occupied) are modelled directly and the occupancy itself is calculated from the previous time period's occupancy status and the transition parameters [17].

We were interested in understanding which factors were associated with higher or lower RABV occupancy in this cross-border region. Given the examination of raccoon RABV occupancy, we hypothesised that the density of raccoons likely impacts the circulation of RABV and examined habitat characteristics that have been reported to impact raccoon densities (e.g. the per cent cover of deciduous and mixed forests, medium and high human development and cultivated crops as summarised in [27]), as well as the mean elevation in each grid cell. The proportion of deciduous and mixed forest cover and evergreen forest cover were strongly correlated with the elevation, therefore we omitted elevation from final analyses. As RABV incidence varies seasonally and across time, we included temporal effects. A key impact of interest was the relationship between RABV occurrence and management activities. In the study area, the primary management methods used were ORV and TVR. We included a covariate for the log number of raccoons in each grid cell captured for TVR. We used the log to account for a potential diminishing effect of vaccinating a single additional raccoon after many have been vaccinated (e.g. the first vaccinated raccoon may have a larger impact on RABV occurrence than the 301st raccoon vaccinated). We also used an indicator covariate for whether ORV management was active for each year within each grid cell within the study. Cells with ORV for a given year were considered ‘managed’ areas, whereas sites without ORV in a given year were considered ‘unmanaged’ areas. As the ORV area is aiming to prevent the spread of raccoon RABV northwards in this region, the indicator for ORV also distinguishes between areas south of the management area (where RABV is enzootic) and areas north of the management area (where RABV may be absent). This Bayesian hierarchical model was implemented using a custom Markov chain Monte Carlo code written in R [28]. Model convergence was assessed graphically and using Gelman–Rubin statistics [29]. The full posterior distribution and conditional distributions are provided in Supplementary S1, similar to Davis *et al*. [16]. We used the occupancy-adjusted version of the area under the curve (AUC) statistic to assess model fit [30].

As described earlier, occupancy is a derived parameter from the estimated occupancy at the previous time step and the transition rates. To refine examination of the impacts of management and other factors on RABV occupancy, we conducted a post-hoc analysis using the derived occupancy estimates from the dynamic occupancy model as the response variable in a generalised linear model framework. Occupancy probability, the response variable, was bounded between zero and one, and therefore we used a beta distribution. We conducted this using the function betareg, in the package ‘betareg’ [31] in program R [28]. In this context, we examined the relationship between occupancy and variability across years and an interaction with region (north of the management area, ORV managed areas and areas south of the management area which are enzootic for RABV), and the lasting effect of management by examining the time since an area was last managed with ORV. We also included seasonal, neighbour and habitat effects. To specifically evaluate impacts of different management decisions within managed areas, we examined the relationship between RABV occupancy and the number of years an area was actively managed using ORV, the impact of the different vaccine bait types (V-RG and ONRAB), baiting densities (typically 75 or 150 baits/km^2^) and the log number of animals that were parenterally vaccinated using TVR, while accounting for habitat variability. We allowed for non-linear relationships between covariates and the response variable using splines (from the ‘splines2’ package [32]). We used a hurdle-type approach to model the overall occupancy probability as a function of the habitat, general indicator of management and temporal variables, and then a more focal analysis on the management methods in managed areas only. We compared models using the second-order Akaike information criterion (AIC); lower AIC values are better and models within 2 AICc of the lowest AICc were considered competitive [33].

We used estimates of occupancy and detection from the dynamic occupancy model to jointly estimate elimination probability across space and time, and to determine the number of samples needed to ensure elimination after the last time period analysed in our study. We examined how many negative samples would need to be collected per year per site to improve the occupancy estimates in these areas to help provide guidance on future sampling that should be conducted to better understand RABV risk corridors.

## Results

Within our study area during 2008–2018, we sampled 15 978 raccoons, 9571 from Québec and 6407 from the USA. There were 27 RABV-positive raccoons sampled from Québec and 622 from the USA. In addition, there were other species that were confirmed positive with the raccoon variant of RABV (Table 1). The greatest number of RABV-positive samples was observed during the first year of our study in 2008 (*n* = 161, representing a raw positivity rate of 7%), whereas during the last year of our study we observed one of the fewest numbers of RABV positives (*n* = 56, representing a raw positivity rate of 3%). The number of samples collected by 100 km^2^ grid cell varied from 0 to 836, with more samples taken the north of the study area compared to south (Supplementary Fig. S2.1)

**Table 1.** Counts of total number of individuals sampled and the number of rabies positives are shown by species, country and year for the study area from 2008 to 2018 in the northeastern USA and in southern Québec, Canada

Species	2008	2009	2010	2011	2012	2013	2014	2015	2016	2017	2018	Total
Canada												
Raccoon	1744 (26)	1980 (0)	865 (0)	609 (0)	656 (0)	596 (0)	508 (0)	538 (1)	504 (0)	875 (0)	696 (0)	9571 (27)
Skunk	6	2	0	0	0	0	0	0	0	0	0	8
USA												
Raccoon	680 (97)	264 (57)	262 (39)	290 (40)	287 (74)	715 (59)	382 (47)	613 (46)	875 (67)	914 (49)	1125 (47)	6407 (622)
Skunk	21	14	11	8	31	27	4	9	11	20	4	160
Fox	8	4	1	4	2	7	7	2	6	6	3	50
Domestic cat	1	2	2	5	5	2	1	0	0	1	1	20
Groundhog	0	0	1	0	2	1	2	0	2	1	0	9
Bobcat	0	0	0	0	0	2	0	0	0	1	1	4
Cattle	0	0	0	1	2	0	0	1	0	0	0	4
Goat	0	2	0	1	0	0	0	0	0	0	0	3
Sheep	2	0	0	0	1	0	0	0	0	0	0	3
Coyote	0	0	0	0	1	0	0	0	0	0	0	1
Domestic dog	0	0	0	0	0	1	0	0	0	0	0	1
Fisher	0	0	1	0	0	0	0	0	0	0	0	1
Horse	0	0	0	0	0	1	0	0	0	0	0	1

For all species other than raccoons (*P. lotor*), only specimens that were positive for the raccoon variant of rabies were included in the study, therefore only one number is shown. For raccoons, the total number of raccoons sampled are shown with the total number of rabies positives in parentheses. The other species include striped skunks (*Mephitis mephitis*), foxes (*Vulpes vulpes* and *Urocyon cinereoargenteus*), domestic cats (*Felis catus*), groundhogs (*Marmota monax*), bobcats (*Lynx rufus*), cattle (*Bos taurus*), goats (*Capra hircus*), sheep (*Ovis aries*), coyote (*Canis latrans*), domestic dog (*Canis lupus familiaris*), fisher (*Pekania pennanti*) and horse (*Equus caballus*).

**Fig. 2.**
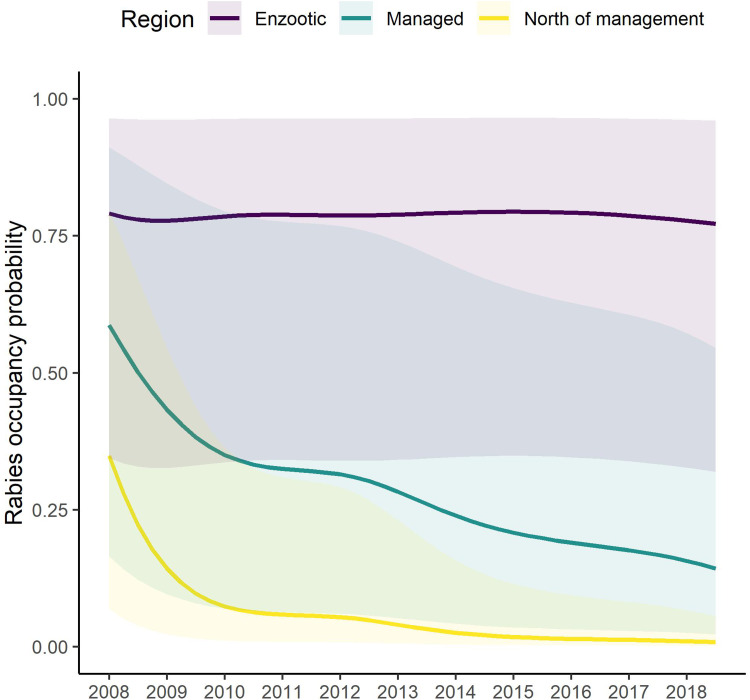
Raccoon rabies occupancy probability across time (year) by management area: south of the managed area which is enzootic for raccoon rabies (purple); within the managed area (teal); or north of the managed area (yellow). These estimates are from the post-hoc occupancy examination. Shaded regions are the 95% prediction intervals.

**Fig. 3.**
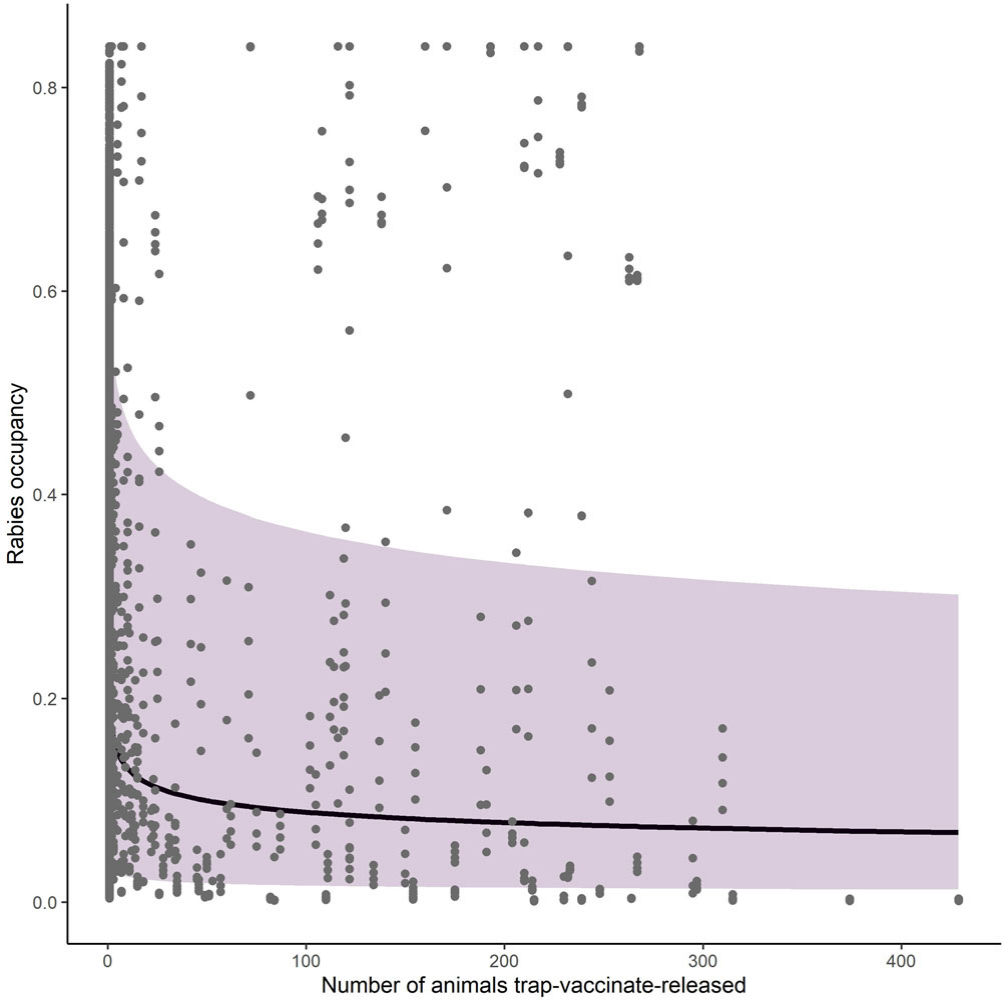
Relationship between the number of animals vaccinated during TVR efforts within a 100 km^2^ grid cell and raccoon rabies occupancy. Shaded region shows the 95% prediction interval. The data on the number of animals trap-vaccinate-released in each grid cell and their respective RABV occupancy probabilities are shown as grey dots.

The dynamic occupancy model performed well using the occupancy-adjusted AUC metric (0.82). Management activities were the largest driver of RABV occupancy within our study area (Supplementary Table S2.1). Particularly for ORV management, unmanaged areas that were south of the management zone (enzootic areas) had high RABV occupancy probabilities (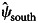 = 0.85, s.e. = 0.03), managed areas within the zone had low RABV occupancy probabilities (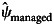 = 0.18, s.e. = 0.21) and areas north of the management zone had occupancy probabilities near zero (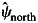 = 0.02, s.e. = 0.14). RABV occupancy remained constant during 2008–2018 in the enzootic areas but substantially decreased across time in both the managed areas and areas north of the management zone (Fig. 2). TVR management also had a strong impact on raccoon RABV occupancy, where the probability of raccoon RABV occupancy decreased per the log number of animals parenterally vaccinated within a grid cell (*β*_TVR_ = −0.23, s.e. = 0.01; Fig. 3).

Raccoon RABV occupancy was also influenced by the occupancy status of neighbouring grid cells. The proportion of neighbouring cells that were infected with raccoon RABV in a given time period had a positive influence on the probability a given cell would be infected (Supplementary Table S2.1). For example, the probability of a cell being infected in managed areas if the cell had no infected neighbours would be 0.22 (95% credible interval (CI) 0.21–0.23) compared to 0.58 (95% CI 0.56–0.60) if all of its neighbours were infected. We also examined habitat effects on RABV occupancy. Habitat coverage was unequally distributed by ORV management location (enzootic areas, ORV managed areas or areas north of the ORV management zone; Supplementary Fig. S2.2). Habitat relationships we observed tended to correlate with the ORV management where they were dominant (Supplementary Table S2.1).

**Fig. 4.**
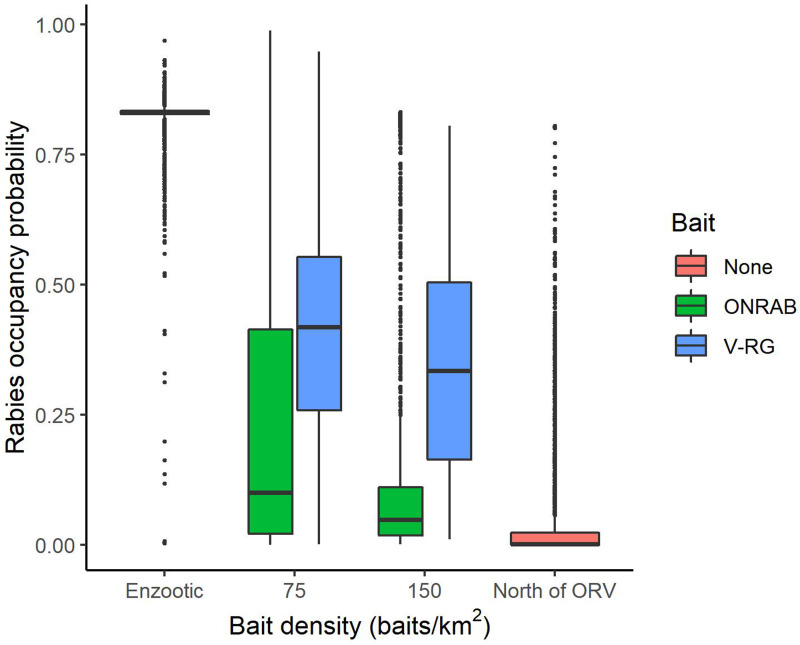
Probabilities of raccoon rabies occupancy in areas south of ORV management (enzootic areas), areas that are managed with two ORV vaccine baits (ONRAB and RABORAL V-RG^®^) at 75 and 150 baits/km^2^, and areas north of ORV management (no baiting, free of raccoon rabies). The solid middle bar is the median, the box represents the middle 50% of the data, the vertical line represents 1.5 times the interquartile range, all points are outside 1.5 times the interquartile range.

To better understand influential factors within management areas, we examined several strategies by restricting the analysis only to areas that were actively managed (Supplementary Table S2.2). Estimated RABV occupancy within managed areas varied by vaccine bait type (V-RG *vs.* ONRAB) and by bait density (Fig. 4). The longer continuous management was conducted, the lower the probability of RABV occupancy with an average reduction of 0.044 percentage points per year (Supplementary Table S2.3).

The probability of RABV detection (across the entire study area) was highest for samples from animals that were strange acting, found dead or from public health surveillance samples (Table 2). The majority (58%) of samples in the USA came from the public health surveillance (*n* = 3729). The other-known and road-kill samples were the next most common samples from the USA (14% and 11% of samples, respectively) and were the two most common samples from Québec (20% other-known and 49% road-kill) yet demonstrated low RABV detection probabilities in both the USA and Québec (Table 2).

**Table 2.** Detection probabilities by surveillance category for the raccoon variant of RABV from the northeastern USA and southern Québec, Canada from 2008 to 2018

				Canada		USA	
Category	Detection	95% CI	Samples	Negative	Positive	Negatives	Positive
Strange acting	0.25	(0.18–0.31)	1561	1020	7	472	62
Found dead	0.18	(0.09–0.30)	920	835	8	69	8
Road-kill	0.05	(0.02–0.08)	5358	4668	3	665	22
Surveillance trapped	0.03	(0.02–0.06)	1183	781	6	392	4
Other-known	0.00	(0.00–0.01)	2832	1952	0	879	1
Unknown	0.47	(0.32–0.65)	151	47	0	79	25
Public health	0.25	(0.23–0.28)	3973	241	3	3229	500
Other species	1.00	(0.99–1.00)	265	0	8	0	257

The 95% credible intervals are provided, along with the number of samples across the entire study area as well as the number of negatives and positives by Canada and the USA.

**Fig. 5.**
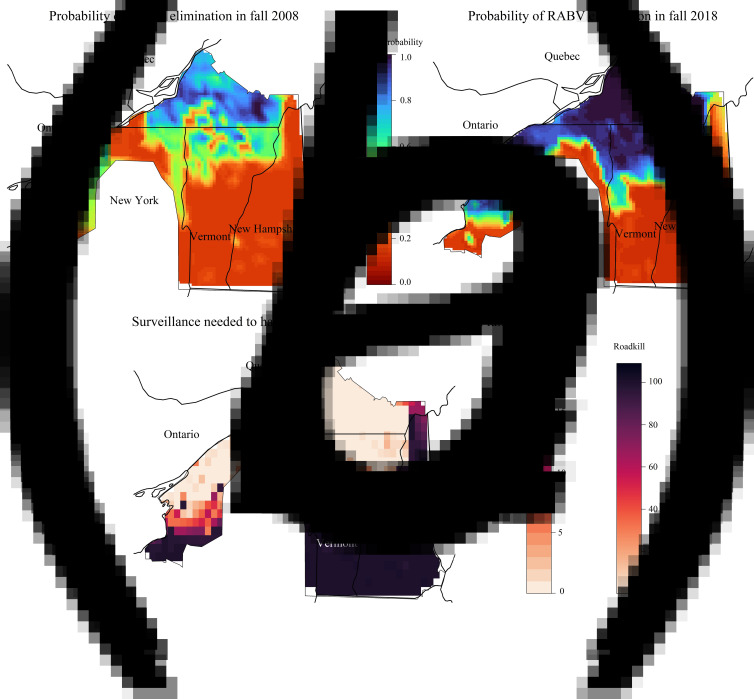
Probability of RABV elimination during (*a*) the first autumn (2008) in the study area and (*b*) the last time point in the study (autumn 2018) and (*c*) the surveillance needed based on the RABV occupancy probability at the last time point in the study (autumn 2018). The minimum number of samples needed varies across space and depending on the surveillance category used. The two scales show the surveillance needs for samples coming from strange acting animals (higher probability of detection) and road-kill animals (lower detection probability). Note that elimination probabilities in northern New Hampshire and the edges of the Adirondacks in New York are likely biased low due to low sampling in these areas due to low expected raccoon densities in these areas.

Raccoon RABV elimination probability increased over time (Figs 5*a* and 5*b*). In the autumn of 2008, there were 11 grid cells with at 95% or greater probability of elimination (10 in Canada and one in the USA). In the autumn of 2018, there were 200 grid cells with a 95% or greater probability of elimination (106 in Canada and 94 in the USA). Elimination probability was the greatest north of areas managed with ORV as expected, particularly throughout southern Québec (Figs 5*a* and 5*b*). Most areas managed with ORV also had greater probabilities of elimination based on the occupancy estimates during the last season sampled in this study (autumn 2018). Within managed areas, cells closer to the enzootic areas had higher RABV occupancy than those closer to the northern ends of the study area (Fig. 5*a*). Areas with RABV detections are shown with a zero probability of elimination (Figs 5*a* and 5*b*). The number of samples needed to have a 95% probability of elimination depends both on the occupancy probability of the cell and the surveillance classification. The greater the probability of RABV detection for a given surveillance method, the fewer the number of samples needed to be confident of elimination (Fig. 5*c*). Very low intensity surveillance would be needed (0–5 samples per cell per season) along the border between the USA and Canada if samples associated with greater detection probability were available (i.e. strange acting, found dead and public health, Fig. 5c). However, if only road-kill, surveillance trapped or other-known samples were available, an increased surveillance effort (15–20 samples per cell) would be needed to be assured of elimination (Fig. 5*c*). In enzootic areas with low expected raccoon densities (e.g. elevation >500 m or with substantial coverage of evergreen forests), like the northern perimeter of the Adirondack State Park in New York and northern New Hampshire, there was little to no surveillance (Fig. 1). These areas are not expected to have high RABV occupancy due to the low densities of raccoons [27]; however, without surveillance in these areas the model could not estimate low rabies occupancy risk in these areas. By generating potential surveillance data, we found that collecting 4–6 samples per cell per year (and if they were all negative) could change the estimated occupancy in enzootic areas which previously lacked surveillance data. Therefore, to better understand the RABV risk in assumed low raccoon density areas, 4–6 samples per cell per year in these areas should be collected annually for testing.

## Discussion

Throughout our cross-border study area, there was a very high probability that Québec was free of raccoon RABV at the end of the study (autumn 2018). Additionally, there were high probabilities of RABV elimination within the USA along the border with Québec in northern New York and Vermont. Our results provide evidence that ORV management was the largest driver of reduced raccoon RABV infection and suggest that intensive management has successfully eliminated RABV in 18 900 km^2^ within 11 years of coordinated management. The success in this study area has resulted in the cessation of ORV management in Québec as of 2021 and the movement of the ORV zone 60 km (37 miles) south within the USA from the Canadian border as of 2022. This large-scale movement of an ORV zone in the northeastern USA is one of the first significant tactical changes to a long standing ORV zone in support of the national strategic goal of raccoon RABV elimination [6]. Dynamic occupancy models as used in this study help provide data-driven recommendations in the event future movement of ORV zones could occur or more intensive management is required in a given area to locally eliminate raccoon RABV (e.g. [24]).

Across the entire study area, management was a primary driver of decreasing RABV occurrence. Both ORV and TVR showed marked declines in RABV occupancy (Figs 2 and 3). By hand-vaccinating 10 raccoons per 100 km^2^ there is an estimated decrease in RABV occupancy in managed areas of 35% (0.22–0.15, Fig. 3). However, returns are diminishing and improvements in RABV occupancy reductions are less dramatic after 50 individuals per 100 km^2^ are hand-vaccinated (Fig. 3). Although TVR programmes are labour intensive, they can be effective in reducing RABV occurrence, especially in localised areas. Areas managed with ORV using ONRAB tended to have lower probabilities of RABV occupancy than those using V-RG. This is supported by previous studies [25] that examined population seroconversion rates of raccoons in areas baited with ONRAB and V-RG and found higher seroprevalence in areas baited with ONRAB compared to V-RG [34, 35]. The impact of bait density was slight, but RABV occupancy tended to decline with higher bait densities for both vaccine bait types, however there was considerable variability across the study area. Similar effects of baiting density on seroconversion have been documented where rabies antibody seroprevalence increased with greater baiting density [10, 13, and 36]. It is important to note that our results are based on an observational study which was not designed to compare vaccine bait types or bait densities in a systematic or controlled fashion. The baiting history of the cells may vary considerably and unequally between bait types and densities so some of our comparisons and conclusions may not be unbiased. A well-designed field experiment would be needed for conclusive statements about the impact of bait type and target density on RABV occupancy in this or other regions.

There were declining trends in RABV occupancy within areas managed by ORV and areas north of the ORV management zones but RABV occupancy probabilities across time in enzootic areas were relatively high and constant (Fig. 2). This result further highlights the need for management to control raccoon RABV, as RABV occupancy does not appear to be declining in the absence of management. In a previous study focused on just the northern parts of New York, Vermont and New Hampshire, Davis *et al*. [16] found a decline in RABV occupancy in managed areas but also found a slight decline in RABV occupancy in the neighbouring enzootic areas. Areas closer to the management zone might benefit from either movement of vaccinated animals into the enzootic area thereby decreasing the number of susceptible animals which helps reduce transmission in those populations, or simply by lowering the RABV pressure by reducing the number of RABV-positive animals that may move into an area. Davis *et al*. [16] examined a smaller enzootic area than our current study. It may be that enzootic areas nearer to management may have a beneficial effect that was not observed at the larger scale of our study. The broader scale of our study and the lower number of samples collected per km^2^ in enzootic areas limited our model's ability to distinguish much variability within the enzootic areas of our study. Davis *et al*. [16] also identified potential risk corridors for RABV spread northwards from the USA into Québec; however that study did not include data from Québec or data from 2018. Risk corridors as defined in that study and as used here are areas with higher RABV occupancy probabilities which may allow RABV spread northwards or areas with lower surveillance and thus greater uncertainty in occupancy probabilities. Our broader study supported the risk corridor in northern Franklin county, NY. However, our results here do not show support for higher risk along the NY–VT border likely due to the high confidence in raccoon RABV elimination within Québec north of this area coupled with the larger distance to the enzootic area within the USA. A study that is more focused on the border region than our current study but one that includes data from both sides of the border may do a better job evaluating risk corridors by reducing the chance of local level patterns to be swamped by the larger regional differences in RABV occurrence.

Understanding host species densities and dynamics are useful to understand pathogen dynamics [37]. There were no detailed raccoon density estimates available across our study area during 2008–2018, therefore, we used habitats as proxies that may relate to raccoon densities and thus may impact the probability of RABV occurrence. However, within our study area the distribution of habitat types varied with the primary driver of RABV occupancy, which was ORV management activities (e.g. cultivated crops were primarily north of the management zone and evergreen forest cover was primarily in enzootic areas). To understand the relationship between habitat cover and RABV occurrence it would be ideal to examine areas just within the enzootic area to not conflate management impacts and habitat. However, as previously mentioned the surveillance efforts in enzootic areas were considerably lower than in management zones or areas north of management zones, and thus there was little power to distinguish differences in RABV occupancy within enzootic areas. This resulted in some areas in our study with high probabilities of raccoon RABV occupancy but without detections of RABV. Primarily these were areas of higher elevation and in evergreen forests in the northern part of New Hampshire and in New York in the Adirondack Mountain region. Raccoon densities tend to be lower at higher elevations and/or in evergreen forests within the eastern USA [27], and thus may not be permissive to RABV perpetuation or spread. The fact that our results suggest a higher RABV occupancy in these areas is counterintuitive. However, without sufficient sampling in these areas our analytical approach was unable to distinguish these sites from other enzootic sites in terms of RABV risk. We attempted to account for this issue to some extent by removing most of the Adirondack Mountain region from the study area due to the lack of surveillance within this higher-elevation area [27, 38]. To understand raccoon RABV infection status in higher-elevation areas, enhanced surveillance is needed. To determine the amount of sampling needed in these regions, we reran the analyses with additional negative only samples, and found that by sampling 4–6 animals per year per 100 km^2^ cell in these areas, we could improve estimates of the RABV risk in high-elevation areas.

Within both managed and unmanaged areas, the occupancy status of neighbouring cells had a positive impact on the probability a cell would be infected with raccoon RABV. Our cell sizes are 10 km × 10 km, based on previous work [25] which aims to balance potential areas of closure of rabies occupancy status (i.e. areas where the RABV occupancy status did not change within a season) and sufficient sampling per cell. Our cells were larger than some previous work examining raccoon RABV dynamics (e.g. [12, 39]). However, our interest was at a broader spatial scale spanning multiple states and into Canada. Our cells are larger than the average home range size of a raccoon; home range sizes are often used as guides for occupancy designs [40]. However, we are modelling the occupancy of RABV in raccoon populations and not individual presence of raccoons, therefore the host home range may be too small to understand broader patterns in population level disease dynamics. Rees *et al*. [41] found that the probability of finding a rabid raccoon was highest within 10 km of the nearest positive case, which supports the idea that the range to understand rabies dynamics is likely larger than the home range size of its host. Additionally, our finding of an important neighbour effect suggests that the cell size we used was reasonable. If these cells had been too large to capture RABV dynamics, a neighbour effect likely would not exist.

Regular enhanced surveillance is a critical component of effective wildlife disease management to determine when wildlife diseases are present and when disease prevention, control or elimination has been reached. Occupancy models estimate detection probability as well as occupancy probability. We used a multi-method approach [26] to simultaneously estimate detection probabilities for multiple surveillance sample types. Surveillance samples with higher detection probabilities (e.g. strange acting, found dead and public health samples) are more efficient at detecting RABV based on the behavioural impacts of this neurologic infection (e.g. acting sick, being aggressive or paralysed [42, 43]). However, these types of samples are often limited by opportunity but also by citizen awareness to report suspect animals (e.g. it is difficult to proactively control the type of samples collected). Surveillance trapping had a relatively low probability of detecting RABV in our study, although it performed better than other-known samples (generally nuisance or healthy animals). Surveillance trapping has been found to be effective at detecting rabid animals in targeted areas after an outbreak [41], but may be more costly and less efficient than road-kill sampling for general surveillance. Road-kill surveys are an intentional surveillance approach to find mesocarnivore [44]. Even though detection probabilities from road-kill samples are slightly lower than that of strange acting or found dead animals, focused standardised efforts via surveys can be proactive as an added advantage over the opportunistic surveillance approaches.

By combining the detection probabilities and occupancy probabilities, it is possible to determine the number of samples that would need to be collected across the study area to be confident that raccoon RABV is eliminated [26]. In areas where there was high confidence in raccoon RABV freedom, such as within Québec and in the USA along the US–Canada border, there would need to be 0–5 road-kill samples per 100 km^2^ cell (fewer samples from strange acting specimens or more samples from nuisance animals). In areas that are managed with ORV but are closer to enzootic areas would require substantially more samples to be confident of RABV freedom in these areas (15–20 road-kill samples or 3–5 samples from strange acting animals). Such estimates can facilitate surveillance programme design for the optimised use of limited resources. In 2022 the ORV management area was moved south from the US–Canada border. While strategic movement of ORV management zones is essential to make progress towards raccoon RABV elimination, continued surveillance effort is necessary to regularly confirm elimination status and ensure long-term maintenance of raccoon RABV-free areas [5]. Application of these surveillance estimates will be particularly useful to adequately evaluate whether elimination in the new zones has been achieved. As well, continued surveillance efforts will provide valuable data to update the occupancy model analyses, thus ensuring that elimination status and resource needs will be evaluated based on latest information for the region.

## Data Availability

The data associated with this manuscript will be available at the US Forest Service Research Data Archive upon acceptance.

## Supplementary material

For supplementary material accompanying this paper visit http://doi.org/10.1017/S095026882300047X. click here to view supplementary material
