# Supplementary material for: Raccoon rabies control and elimination in the northeastern USA and southern Québec, Canada
Source: Epidemiol Infect. 2023 Mar 22;151:e62. doi: 10.1017/S095026882300047X (PMC10126900; doi:10.1017/S095026882300047X)
Supplement: Supplementary file 1 [file hygsup.zip › S095026882300047Xsup001.pdf]

Supplemental Information S1: Model, Joint Distribution, and Conditional Distributions for "Raccoon rabies control and elimination in the northeastern U.S. and southern Québec, Canada"

Amy J. Davis, Marianne Gagnier, Ariane Massé, Kathleen M. Nelson, Jordona D. Kirby, Ryan Wallace, Xiaoyue Ma, Christine Fehlner-Gardiner, Richard B. Chipman, and Amy T. Gilbert

Dynamic multi-method occupancy model

To determine the local occupancy status of the raccoon variant of the rabies virus (RABV) within our study area, we sampled raccoons from a variety of surveillance methods. The estimated occupancy in grid cell i and at time period t is denoted by ψ_{it}. The true occupancy status (z_{it}) is an unknown latent variable that is informed by the surveillance data. If any raccoons in a site at a time are found to be positive for RABV then the site is occupied ($z_{it} = 1$). If no surveillance method detects RABV presence then z_{it} might be 1 and we failed to detect RABV, or it could mean that the site was unoccupied with RABV and $z_{it} = 0$.

When the site is occupied ($z_{it} = 1$), the probability of detecting RABV (p) can be calculated for each surveillance method (j). Therefore, the number of RABV-positive raccoons sampled (y_{itj}) is a function of the total number of raccoons sampled at that site, time, and method (n_{itj}), and the detection probability by method (p_j).

The initial occupancy (ψ_{i1}) is estimated from an uninformed prior. All subsequent occupancy estimates are conditioned on the previous time steps and the transition parameters of extinction (ϵ_{it}) and colonization (γ_{it}). We modeled extinction with an uninformed prior. We modeled colonization with covariates we expected may influence either raccoon densities or RABV occurrence.

The full model spec is shown below, as well as the joint distribution and conditional distributions used to code the Markov Chain Monte Carlo (MCMC).

$$y_{ijt} = \begin{cases} 0 & , z_{it} = 0 \qquad i = 1, ..., N \quad \text{(grid cell)} \\ & \qquad\qquad\qquad j = 1, ..., J \quad \text{(surveillance method)} \\ Bin(p_j, n_{ijt}) & , z_{it} = 1 \qquad t = 1, ..., T \quad \text{(time period)} \end{cases}$$

$$p_j \sim Beta(\alpha_p, \beta_p)$$

$$z_{i1} \sim Bern(\psi_{i1}) \qquad \psi_{i1} \sim Beta(1,1)$$

$$z_{it}|z_{it-1} \sim Bern(\psi_{it})$$

$$\psi_{it} = (1 - \epsilon_{i,t-1})z_{i,t-1} + \gamma_{it-1}(1 - z_{it-1})$$

$$\epsilon_{it} \sim Beta(\alpha_\epsilon, \beta_\epsilon)$$

$$logit(\gamma_{it}) = X_\gamma \boldsymbol{\beta_\gamma}$$

$$\boldsymbol{\beta_\gamma} \sim Norm(0,1)$$

Joint Distribution

$$[\boldsymbol{z}, \boldsymbol{\psi}, \boldsymbol{\epsilon}, \boldsymbol{\beta_\gamma}, \boldsymbol{p} | \boldsymbol{y}, \boldsymbol{n}, \boldsymbol{X_\gamma}] \propto \prod_{i=1}^{N} \prod_{t=1}^{T} \prod_{j=1}^{J} \left([y_{ijt}, n_{ijt} | p_j]^{z_{it}} 1^{(1-z_{it})} [z_{it}|z_{it-1}] \right) [\psi_{i1}][\epsilon][\boldsymbol{\beta_\gamma}][p_j]$$

$$[\psi_{i1}|\bullet] \propto \prod_{i=1}^{N} [z_{i1}|\psi_{i1}][\psi_{i1}]$$

$$\sim Beta\left(\sum_{i=1}^{N} z_{i1} + 1, \sum_{i=1}^{N} (1 - z_{i1}) + 1\right)$$

for $z_{i,t-1} = 1$

$$[\epsilon|\bullet] \propto \prod_{i=1}^{N}\prod_{t=1}^{T} [z_{it}|z_{i,t-1}][\epsilon]$$

$$\sim Beta\left(\sum_{i=1}^{N}\sum_{t=1}^{T} z_{it} + 1, \sum_{i=1}^{N}\sum_{t=1}^{T} (1 - z_{it}) + 1\right)$$

for $z_{i,t-1} = 0$

$$[\boldsymbol{\beta_\gamma}|\bullet] \propto \prod_{i=1}^{N}\prod_{t=1}^{T} [z_{it}|z_{i,t-1}][\boldsymbol{\beta_\gamma}]$$

Metropolis-Hastings

$$MHratio = \left(\frac{\prod_{i=1}^{N}\prod_{t=1}^{T} Bern(\boldsymbol{X\beta_\gamma}^*|z_{it})}{\prod_{i=1}^{N}\prod_{t=1}^{T} Bern(\boldsymbol{X\beta_\gamma}|z_{it})}\right) \times \left(\frac{Norm(\boldsymbol{\beta_\gamma}^*, 0, 1)}{Norm(\boldsymbol{\beta_\gamma}, 0, 1)}\right)$$

for $y_{ijt} = 0$

$$[z_{it}|\bullet] \propto \prod_{j=1}^{J} \left([y_{it}, n_{it}|p_j]^{z_{it}} 1^{(1-z_{it})}\right) [z_{it}|z_{it-1}, z_{it+1}]$$

$$\sim Bern\left(\frac{\prod_{j=1}^{J} (1 - p_j)\psi^*_{it}}{\prod_{j=1}^{J} (1 - p_j)\psi^*_{it} + (1 - \psi^*_{it})}\right)$$

ψ^* is dependent on the status of z_{t-1} and z_{t+1} described in the conditions below

$$[\psi^*_{1,1}] = \frac{(1-\epsilon_{t-1})(1-\epsilon_t)}{(1-\epsilon_{t-1})(1-\epsilon_t) + \epsilon_{t-1}\gamma_t}$$

$$[\psi^*_{1,0}] = \frac{(1-\epsilon_{t-1})\epsilon_t}{(1-\epsilon_{t-1})\epsilon_t + \epsilon_{t-1}(1-\gamma_t)}$$

$$[\psi^*_{0,1}] = \frac{\gamma_{t-1}(1-\epsilon_t)}{\gamma_{t-1}(1-\epsilon_t) + (1-\gamma_{t-1})\gamma_t}$$

$$[\psi^*_{0,0}] = \frac{\gamma_{t-1}\epsilon_t}{\gamma_{t-1}\epsilon_t + (1-\gamma_{t-1})(1-\gamma_t)}$$
