# Supplementary material for: Raccoon rabies control and elimination in the northeastern USA and southern Québec, Canada
Source: Epidemiol Infect. 2023 Mar 22;151:e62. doi: 10.1017/S095026882300047X (PMC10126900; doi:10.1017/S095026882300047X)
Supplement: Supplementary file 1 [file hygsup.zip › S095026882300047Xsup002.pdf]

Supplemental information S2. Supplemental tables and figures for the raccoon rabies occupancy analysis of the northeastern U.S. and Québec, Canada from 2008-2018.

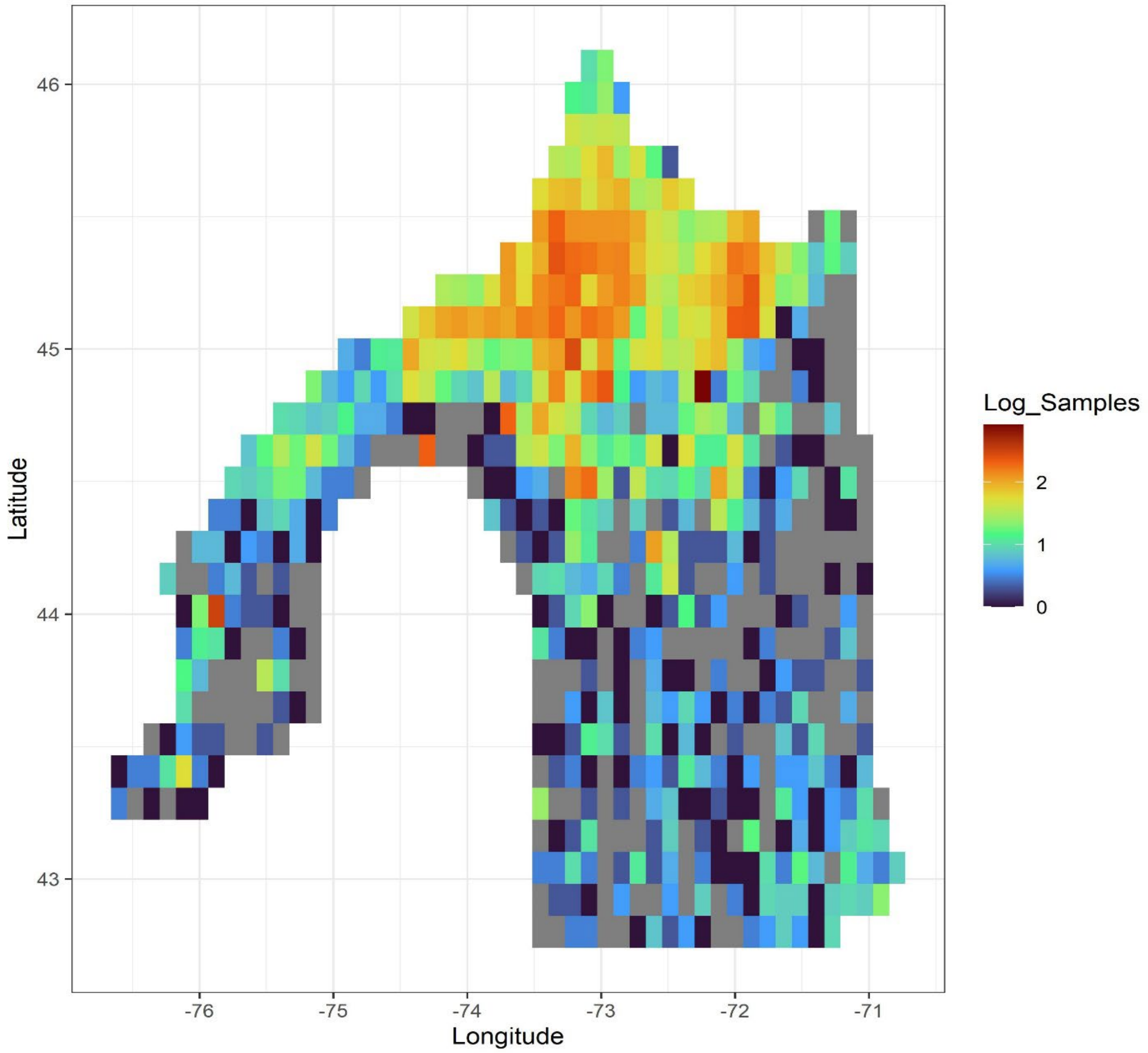

Figure S2.1. The log base 10 number of samples collected by site (100km^2 grid cell) from 2008-2018 within the study area in the northeastern U.S. and southern Québec to examine raccoon rabies occupancy.

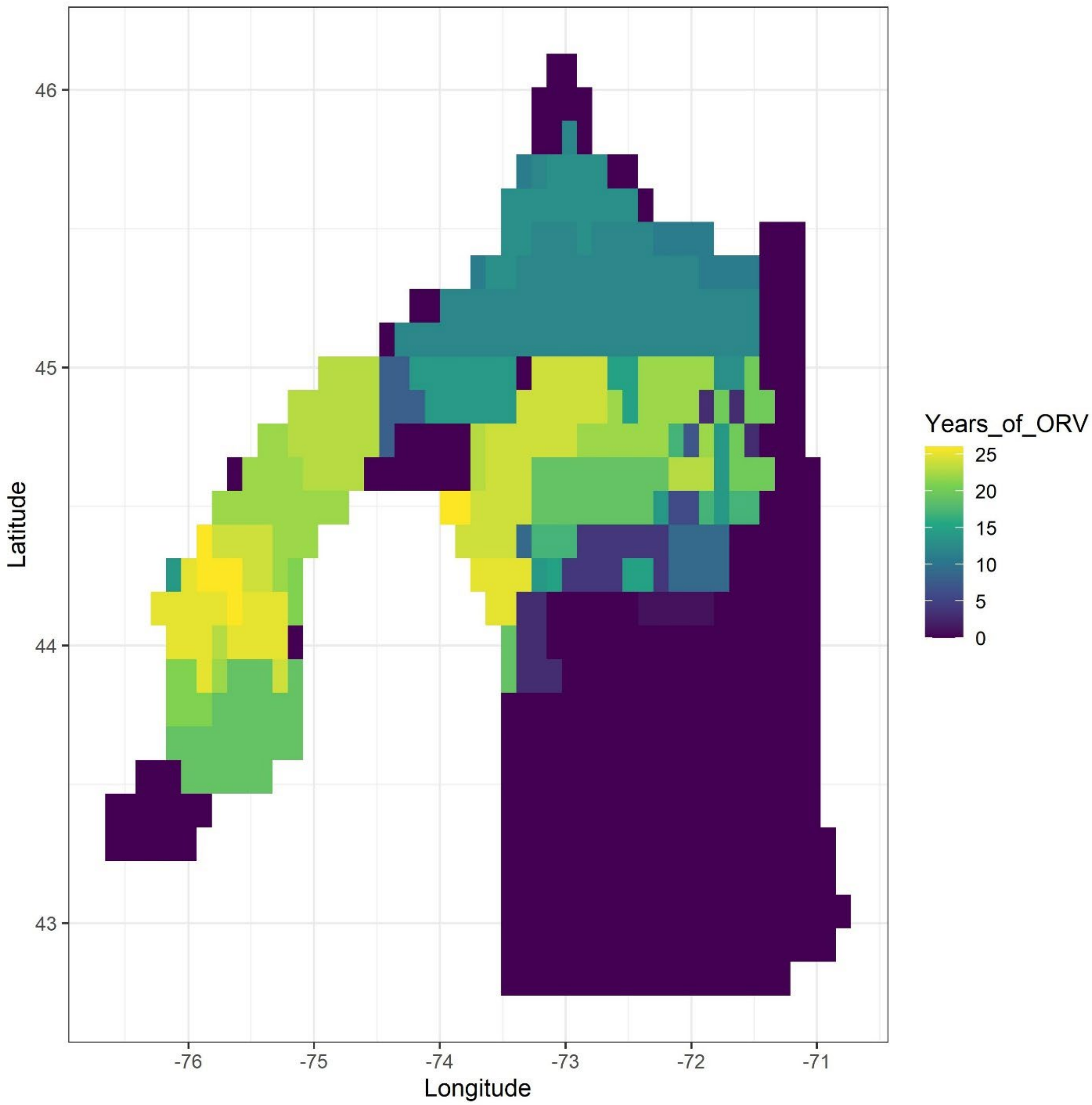

Figure S2.2. The cumulative number of years where oral rabies vaccination management was conducted by site (100km^2 grid cell) from 2008-2018 within the study area in the northeastern U.S. and southern Québec.

Table S2.1. Parameter estimates, standard errors, z-values, and p-values for the most supported model from the post-hoc examination of the entire study area. The covariates are defined as: time = season by year sequence, DFMF = percent cover of deciduous and mixed forests, EVF = percent cover of evergreen forests, Hay = percent cover of hay and pasture areas, Shrub = percent cover of shrub and scrub cover, MedHi = percent cover of medium and high human development areas, WT = percent cover of wetlands, tvr = scaled number of animals trapped-vaccinated-released within the site, and Neighbs = the proportion of neighbors infected with RABV. All references to 'bs' refer to the basis function splines, the 'df' is the degree of freedom for the splines.

Covariate	Estimate	Std. Error	z value	Pr(>|z|)
(Intercept)	0.25	0.03	8.43	0.00
bs(time2, df = 7)1	-0.49	0.05	-9.83	0.00
bs(time2, df = 7)2	-1.25	0.04	-30.53	0.00
bs(time2, df = 7)3	-0.96	0.04	-22.78	0.00
bs(time2, df = 7)4	-1.62	0.04	-42.14	0.00
bs(time2, df = 7)5	-1.83	0.05	-38.64	0.00
bs(time2, df = 7)6	-1.94	0.05	-42.78	0.00
bs(time2, df = 7)7	-2.14	0.04	-54.19	0.00
ORVNorth	-0.99	0.03	-32.26	0.00
DFMF	-0.20	0.03	-8.03	0.00
EVF	1.44	0.05	26.52	0.00
Hay	-3.96	0.36	-10.94	0.00
Shrub	2.78	0.16	17.59	0.00
MedHi	-0.46	0.07	-7.11	0.00
WT	1.02	0.07	13.96	0.00
tvr	-0.23	0.01	-32.06	0.00
Neighbs	1.62	0.03	54.28	0.00
bs(time2, df = 7)1:ORVNorth	-0.35	0.07	-5.39	0.00
bs(time2, df = 7)2:ORVNorth	-1.33	0.05	-24.86	0.00
bs(time2, df = 7)3:ORVNorth	-0.90	0.05	-16.46	0.00
bs(time2, df = 7)4:ORVNorth	-1.65	0.05	-32.85	0.00
bs(time2, df = 7)5:ORVNorth	-1.85	0.06	-29.73	0.00
bs(time2, df = 7)6:ORVNorth	-1.86	0.06	-31.64	0.00
bs(time2, df = 7)7:ORVNorth	-2.03	0.05	-40.29	0.00

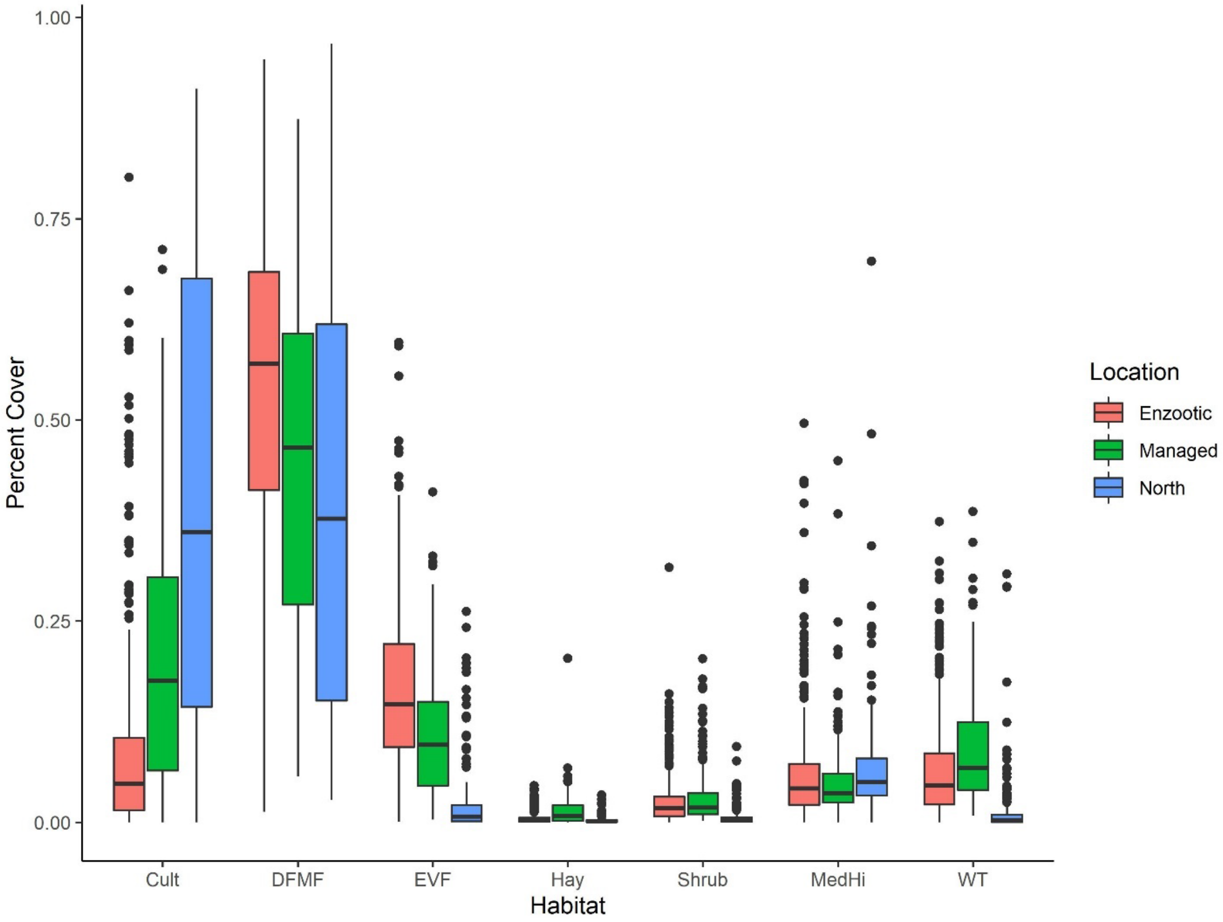

Figure S2.3. Distribution of percent cover by habitat type and location: areas enzootic for raccoon rabies virus (red), areas managed by oral rabies vaccination (green), and, areas north of the management zones (blue). The habitat types are: Cult = cultivated crops, DFMF = deciduous and mixed forest, EVF = evergreen forest, Hay = hay and pastureland, Shrub = shrub and scrub, MedHi = medium and high human development, and WT = wetlands. The solid middle bar is the median, the box represents the middle 50% of the data, the vertical line represents 1.5 times the interquartile range, all points are outside 1.5 times the interquartile range.

Table S2.2. Table of model results for the RABV occupancy post-hoc analysis of the managed areas of the study area. All models include the all habitat covariates including percent cover of deciduous and mixed forests, evergreen forests, hay and pasture areas, shrub and scrub cover, medium and high human development areas, and wetlands. Other parameters are defined as: TVR = log number of animals trapped-vaccinated-released within the site, YrsORV = the number of continuous years ORV baiting has occurred in the site, Bait = the indicator variable for if Raboral V-RG or ONRAB was used as the ORV bait type, and DenGroup = the indicator variable for if the target bait density was 150 baits/km^2 compared to 75 baits/km^2. Interactions are shown with asterisks. All references to ‘bs’ refer to the basis function splines, the ‘df’ is the degree of freedom for the splines. K refers to the number of parameters for each model. The Delta AICc shows the difference in AICc values from the top model. The AICc of the top model is -10167.9. LL is the log likelihood for each model.

Model	K	Delta_AICc	LL
bs(TVR,df=3)+Bait*bs(YrsORV,df=3)+DenGroup*Bait	20	0	5104.01
TVR+Bait*bs(YrsORV,df=3)+DenGroup*Bait	18	15.51	5094.24
bs(TVR,df=3)+Bait+bs(YrsORV,df=3)+DenGroup*Bait	17	15.96	5093.01
bs(TVR,df=3)+Bait*YrsORV+DenGroup*Bait	16	18.37	5090.81
bs(TVR,df=3)+Bait*bs(YrsORV,df=3)	18	22.1	5090.95
bs(TVR,df=3)+Bait+YrsORV+DenGroup*Bait	15	23.7	5087.13
TVR+Bait+bs(YrsORV,df=3)+DenGroup*Bait	15	28.25	5084.86
bs(TVR,df=3)+Bait*YrsORV	14	32.43	5081.77
TVR+Bait*YrsORV+DenGroup*Bait	14	34.48	5080.74
bs(TVR,df=3)+Bait+bs(YrsORV,df=3)	15	37.13	5080.42
TVR+Bait+YrsORV+DenGroup*Bait	13	38.67	5077.64
TVR+Bait*bs(YrsORV,df=3)	16	39.05	5080.46
bs(TVR,df=3)+Bait+YrsORV	13	41.7	5076.13
TVR+Bait*YrsORV	12	49.65	5071.15
TVR+Bait+bs(YrsORV,df=3)	13	50.66	5071.65
TVR+Bait+YrsORV	11	57.68	5066.13
YrsORV	9	1195.36	4495.29
TVR	9	2602.43	3791.75
DenGroup	9	2625.09	3780.42
Habitat only	8	2771.98	3705.97

Table S2.3. Parameter estimates, standard errors, z-values, and p-values for the most supported model from the post-hoc examination of the managed areas of the study area (see Table S2.2). The covariates are defined as: DFMF = percent cover of deciduous and mixed forests, EVF = percent cover of evergreen forests, Hay = percent cover of hay and pasture areas, Shrub = percent cover of shrub and scrub cover, MedHi = percent cover of medium and high human development areas, WT = percent cover of wetlands, TVR = log number of animals trapped-vaccinated-released within the site, YrsORV = the number of continuous years ORV baiting has occurred in the site, BaitV-RG = the indicator variable for if Raboral V-RG was used as the ORV bait type compared to ONRAB, and DenGroup150 = the indicator variable for if the target bait density was 150 baits/km^2 compared to 75 baits/km^2. Interactions are shown with asterisks. All references to 'bs' refer to the basis function splines, the 'df' is the degree of freedom for the splines.

Covariate	Estimate	Std. Error	z value	Pr(>|z|)
(Intercept)	-0.49	0.05	-8.98	< 2e-16
DFMF	-0.91	0.05	-17.75	< 2e-16
EVF	1.68	0.15	11.40	< 2e-16
Hay	-3.94	0.56	-7.06	0.00
Shrub	4.73	0.37	12.82	< 2e-16
MedHi	-0.66	0.15	-4.36	0.00
WT	0.89	0.14	6.38	0.00
bs(tvr, df = 3)1	-1.13	0.20	-5.59	0.00
bs(tvr, df = 3)2	-0.63	0.28	-2.28	0.02
bs(tvr, df = 3)3	-1.29	0.15	-8.64	< 2e-16
BaitV-RG	0.49	0.06	8.52	< 2e-16
bs(YrsORV, df = 3)1	-0.93	0.12	-7.56	0.00
bs(YrsORV, df = 3)2	-0.46	0.08	-5.52	0.00
bs(YrsORV, df = 3)3	-1.18	0.06	-20.58	< 2e-16
DenGroup150	-0.13	0.03	-4.73	0.00
BaitV-RG:bs(YrsORV, df = 3)1	0.60	0.16	3.66	0.00
BaitV-RG:bs(YrsORV, df = 3)2	-0.45	0.14	-3.23	0.00
BaitV-RG:bs(YrsORV, df = 3)3	0.10	0.11	0.92	0.36
BaitV-RG:DenGroup150	0.21	0.06	3.84	0.00
